# Healthcare Providers' Adherence to COVID-19 Prevention and Control Practices in Health Records and Information Management, Ghana

**DOI:** 10.1155/2024/8862660

**Published:** 2024-07-05

**Authors:** Richard Okyere Boadu, Kwame Adu Okyere Boadu, Nathan Kumasenu Mensah, Godwin Adzakpah, Fortune Afaglo, Rosemary Bermaa Abrefa, Emmanuella Aryee, Nancy Gyamena Botwe, Dinah Baiden-Amissah, Dennis Bless Ashiavor, Larry Lee Mensah, Lovemond Kojo Asamoah, Judith Obiri-Yeboah

**Affiliations:** ^1^ Department of Health Information Management School of Allied Health Sciences College of Health and Allied Health Sciences University of Cape Coast, Cape Coast, Ghana; ^2^ School of Medicine and Dentistry College of Health Sciences Kwame Nkrumah University of Science and Technology, Kumasi, Ghana; ^3^ Health Plus Clinic, Tarkwa, Ghana

## Abstract

**Background:**

The impact of contracting coronavirus on healthcare providers (HCPs) affects their ability to combat the infection. The virus can be transmitted through droplets from sneezing, coughing, and yelling, making it essential for HCPs to plan ahead when dealing with patients with respiratory symptoms. The need to assess healthcare providers' perceived adherence to COVID-19 Prevention and Control Practices (PCP) in Health Records and Information Management is vital for optimizing healthcare operations and ensuring the safety of both patients and providers. This study assesses healthcare providers' perceived adherence to COVID-19 PCP in Health Records and Information Management. *Subjects and Method*. A cross-sectional survey was conducted to collect data from 1268 HCPs working in eight randomly selected hospitals across five regions in Ghana. The survey was carried out from May 15, 2022, to August 13, 2022. Simple random sampling was used to choose these eight facilities from a total of 204 hospitals. Within each facility, HCPs from various departments were selected using simple random sampling. The EpiInfo 7 software's StatCalc tool was used to choose a total sample size of 1268 from an estimated 4482 HCP-PR from the eight hospitals. Compliance with COVID-19 PCP was assessed using a 3-point scale, ranging from one (Yes always) to three (No). Cronbach's alpha reliability coefficient was used to examine the statistical reliability of the variables in the dataset. Cronbach's alpha was 0.73 overall, suggesting strong reliability. Bartlett's test for equal variances was used for comparative analysis of health facility and overall mean COVID-19 PCP in different areas of health facilities. IBM SPSS (version 23) statistical software was used for the data analysis process.

**Results:**

A total of 1268 HCP-PR participated in the survey, resulting in a 99.6% response rate. Findings reveal that 760 healthcare professionals who handle patients' records (HCP-PR), constituting 60%, consistently followed COVID-19 protocols in the registration and clinic preparation zones. Another 390 individuals (30.7%) adhered to these protocols occasionally, while 119 (9.4%) failed to comply. Similarly, in the filing area, 739 respondents (58.3%) consistently adhered to COVID-19 protocols, 358 (28.3%) occasionally did so, and 170 (13.4%) did not follow the protocols at all. Regarding handling health records cautiously, 540 participants (42.5%) always did, 448 (35.3%) did so sometimes, and 280 (22.2%) neglected these precautions. Additionally, 520 respondents (41.0%) consistently followed COVID-19 precautions when handling computers and other equipment, 393 (31.0%) did so occasionally, and 355 (28.0%) did not adhere to these precautions.

**Conclusion:**

The majority of respondents showed good compliance with COVID-19 protocol in the registration and clinic preparation areas. However, in the filing area, just over four out of every seven respondents consistently adhered to COVID-19 PCP. Additionally, four out of every seven participants did not comply with COVID-19 PCP when handling patients' records. Analysis reveals diverse adherence to COVID-19 PCP, and statistical tests show variable performance, highlighting standout health facilities.

## 1. Introduction

Coronavirus disease 2019 (COVID-19) is an infectious disease caused by a newly discovered coronavirus, and the World Health Organization (WHO) declared it a pandemic, representing a global crisis that has spread to every continent except Antarctica. In response to the high risk of transmission, many governments worldwide have taken measures, such as temporarily closing schools, colleges, and universities to contain the spread of COVID-19 [1]. When medical professionals contract COVID-19, it not only affects their physical health but also impacts their attitude and ability to fight the infection [2]. Maintaining a social distance of 1.5 to 2 meters between individuals is an effective measure to prevent the spread of respiratory diseases [3–6]. The virus spreads through air droplets, which can be produced through sneezing, coughing, and yelling [7]. To prevent infection spread, various measures are recommended, including maintaining distance from others, wearing masks, frequent handwashing, and using alcohol-based sanitizers [7, 8]. Proper planning and precautions are crucial when handling patients with acute respiratory symptoms, such as maintaining a minimum contact distance of 2 meters and ensuring COVID-19-positive patients, wear surgical masks [9, 10].

Healthcare providers at the records unit are also required to wear appropriate personal protective equipment (PPE) while handling records, and they should follow infection control and safety precautions to safeguard themselves and their patients [3, 11]. Patients suspected of having COVID-19 should be promptly separated [12]. Hand hygiene is vital for healthcare professionals, both before and after interacting with patients, handling potentially infectious objects, and using PPE [5]. The use of alcohol-based hand sanitizers or soap and water is recommended for effective hand hygiene [5, 13].

Studies have shown that viruses can persist on surfaces for extended periods, highlighting the importance of regular handwashing after contact with potentially contaminated objects [14, 15]. Healthcare providers must wash their hands before and after engaging with patients or touching equipment that may be contaminated [16, 17]. Despite the emphasis on adherence to various COVID-19 procedures, healthcare providers responsible for managing patient health records have received relatively less attention [18]. Therefore, it is crucial for healthcare professionals, as well as anyone handling health data, to closely follow recommended COVID-19 infection prevention and control practices.

In Ghana's COVID-19 treatment centers, healthcare workers play an integral role in managing COVID-19 cases, exposing them to constant contact with SARS-CoV-2 [19]. Failure to adhere to recommended infection prevention and control (IPC) measures can potentially lead to COVID-19 virus infection. This study is crucial for safeguarding public health, ensuring the safety of healthcare workers and patients, maintaining data integrity, optimizing resource allocation, and contributing to global efforts in combating the COVID-19 pandemic [19–22]. Research in this area provides actionable insights for improving healthcare practices and pandemic response strategies in Ghana and beyond. The study's importance lies in its potential to provide nuanced insights into the dynamics of healthcare providers' perceived adherence to COVID-19 Prevention and Control Practices in Health Records and Information Management, leading to targeted improvements in healthcare practices and public health outcomes. This study assesses healthcare providers' perceived adherence to COVID-19 Prevention and Control Practices in Health Records and Information Management.

## 2. Research Methods

### 2.1. Study Design

A cross-sectional design was used to collect data from HCPs working in eight randomly selected hospitals across five regions in Ghana. In this study, 1268 people who work with patient data or information in the chosen hospital (regardless of their age, gender, location, affiliation, degree of fitness, intellectual ability, etc.) are involved. The study included HCP-PR who were chosen at random and who gave their informed agreement to take part.

### 2.2. Study Population

**Figure 1:**
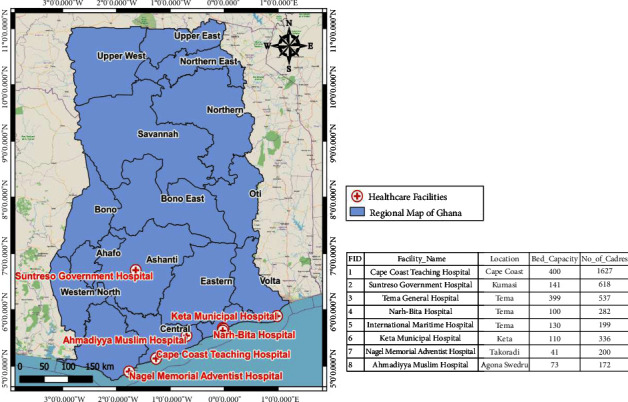
Brief background of the selected hospitals.

HCP-PR in the eight chosen hospitals from five regions of Ghana makes up the study population. From a sample frame of 204 hospitals in the Ashanti, Greater Accra, Central, Western, and Volta regions, eight facilities were chosen at random. Because of their proximity to one another for data collection, these areas were specifically chosen and included in the study. The list of facilities includes the Cape Coast Teaching Hospital (CCTH), Suntreso Government Hospital (SGH), Tema General Hospital (TGH), Narh-Bita Hospital (NBH), International Maritime Hospital (IMH), Keta Municipal Hospital (KMH), Nagel Memorial Adventist Hospital (NMAH), and Ahmadiyya Muslim Hospital (AMH) as indicated in Figure 1.

### 2.3. Sample Size Determination

The EpiInfo 7 software's StatCalc tool was used to choose a total sample size of 1272 (cluster size = 159) from an estimated 4482 HCP-PR from the eight hospitals (confidence level = 99.99%, expected frequency = 50%, margin of error = 5%, design effect = 1.12, cluster = 8). CCTH = 182, SGH = 156, TGH = 160, NBH = 155, IMH = 150, KMH = 160, NMAH = 154, and AMH = 155 are the estimated sample sizes for the chosen facilities.

### 2.4. Sampling Procedure

At each facility, HCP-PR from the various departments and units were chosen at random for the study. A simple random sample was employed to choose participants to ensure that all eligible workers had an equal opportunity to take part in the study in the selected facilities (note: sample distribution of staff categories in each department was done based on an available statistic that was provided by the management of the hospitals before the commencement of the study). Each department or unit generated a list of staff members with serial IDs in the following order: 001, 002,…, *N*, where *N* is the total number of HCP-PR. The study population was covered by selecting participants based on serial IDs using a random generator (Mobile App). The study only included employees who were chosen and gave their assent voluntarily.

### 2.5. Data Collection and Analysis

The data collection process involved using a standardized questionnaire (filled by respondents through digital apps) comprising two components. The first section focused on sociodemographic information, including gender, age, education, profession, and years of experience. In the second section, respondents were assessed on their adherence to COVID-19 prevention and control practices, as well as specific practices related to various aspects of health records and information management. The Healthcare Provider-Prevention and Control Practices for COVID-19 (HCP-PR) scale was employed in the second section. This scale measured adherence to COVID-19 prevention and control practices specific to registration and clinic preparation areas, filing areas, handling of health records, handling of computers and other equipment, and ongoing COVID-19 education. To ensure the questionnaire effectively addressed each indicator relevant to “managing health records and information management,” it was designed in accordance with the health records and information management guidelines and protocols for SARS-CoV-2 and COVID-19 [23]. Each action statement or item related to these characteristics was evaluated on a scale of one (Yes always) to three (No), allowing participants to indicate the extent to which they followed COVID-19 prevention and control methods rigorously. Cronbach's alpha reliability coefficient was used to examine the statistical reliability of the variables in the dataset. This approach was used to evaluate the survey items' internal consistency [24, 25]. Cronbach's alpha was 0.73 overall, suggesting strong dependability. Individual Cronbach's alpha values for the study's various components were also calculated. The tool's Content Validity Index (CVI) and Content Validity Ratio (CVR) were also calculated.  Table 1 shows the results of the reliability and validity tests. Bartlett's test for equal variances was used for comparative analysis of health facility and overall mean COVID-19 PCP in different areas of health facilities. IBM SPSS (version 23) statistical software was used for the data analysis process.

### 2.6. Ethical Considerations

The Cape Coast Teaching Hospital Ethical Review Committee granted clearance for this study (approval number: CCTHERC/EC/2022/078). Prior to data collection, each participating facility received an introduction letter and an ethical permission letter from the University of Cape Coast's Department of Health Information Management. During the introduction section of the questionnaire, formal verbal consent was obtained from all participants. The researchers introduced themselves to the respondents at each interview point and provided a comprehensive explanation of the study's objectives. Participation in the study was entirely voluntary, and respondents were given the autonomy to decide whether or not to take part. To ensure confidentiality, participants were assured that their identities would remain private.

## 3. Results

### 3.1. Background Characteristics of Respondents

A total of 1268 HCP-PR participated in the survey, yielding a 99.6% response rate. Among them, 715 (56.4%) were females, and the remaining 552 (43.6%) were males (Table 2).

Regarding education, the majority of HCP-PR (41.4%) held a bachelor's degree, followed closely by those with diplomas/degrees (41.3%). Additionally, 79 (6.2%) had a master's degree, 75 (5.9%) had other educational qualifications, 47 (3.7%) had postsecondary education, and 18 (1.4%) possessed a junior high secondary/senior high secondary (JSS/SHS) qualification.

The occupational distribution showed that 551 (43.4%) of the participants were nurses, while 167 (13.4%) were biostatisticians/district/hospital health information officers. Furthermore, 120 (9.5%) were midwives, 68 (5.4%) were physician assistants, 66 (5.2%) were pharmacists, and 49 (3.9%) were doctors. The remaining participants belonged to various roles, such as disease control officers, dispensary technicians, laboratory technologists/technicians, and other facility staff.

In terms of work experience, a significant proportion of HCP-PR (72.6%) had been in their profession for 0–5 years, while 250 (19.7%) had worked for 6–10 years. A smaller percentage (5.3%) had 11–15 years of experience, and only 2.4% had worked for 16 years or more. The mean years of experience were 4.6 years, with an SD of 4.3, ranging from 0 to 43 years. Regarding COVID-19-related health information management training, approximately 717 (56.5%) of the HCP-PR had received some form of training in the last six months before the survey.

### 3.2. PCP at the Registration and Clinic Preparation Areas

In the survey, respondents were asked about various COVID-19 prevention practices they followed in organizing clinics and registration areas. Results showed that 777 (61.3%) of HCP-PR ensured clients maintained a 1- to 2-meter social distance from each other, while 454 (35.8%) did so sometimes, and 37 (2.9%) never did. Regarding face mask usage, 940 (74.1%) of HCP-PR wore them always during registration, 293 (23.1%) wore them sometimes, and 35 (2.8%) never wore them. On wearing hand gloves during registration, 512 (40.4%) claimed they wore them always, 382 (30.1%) seldom wore them, and 374 (29.5%) never wore hand gloves.

In terms of hand sanitization, 963 (75.9%) of respondents claimed they sanitized their hands regularly, 279 (22%) did so but not on a regular basis, and 26 (2.1%) never sanitized their hands. When it came to marking a “red taped” area to indicate the minimum distance between the registration desk and clients, 584 (46.1%) of HCP-PR said they did it always, 400 (31.5%) did it sometimes, and 284 (22.4%) never did.

Moreover, 751 (59%) of respondents made sure there was no physical contact with clients during registration, 448 (35.3%) ensured it occasionally, and 69 (5.4%) did not pay attention to this aspect. When asked about avoiding/minimizing the use of papers between patients and providers, 638 (50.3%) responded affirmatively, while 524 (41.3%) did so sometimes, and 106 (8.4%) never avoided/minimized paper usage. Regarding decontaminating surfaces in contact with patients or their belongings, 842 (66.4%) of HCP-PR admitted doing it always, 372 (29.3%) did it seldom, and 54 (4.3%) never did. Additionally, participants were asked if they disinfected computer keyboards, mice, door handles, preparation tables, pens, and so on before and after usage. 829 (65.4%) claimed to comply always, and 355 (28.0%) did so sometimes, while 84 (6.6%) did not follow this practice. In summary, 760 (60%) of HCP-PR always adhered to COVID-19 protocol in the registration and clinic preparation areas, and 390 (30.7%) did so sometimes, while 119 (9.4%) did not adhere to the protocol.

The comparative analysis shows that there is significant variation in health facility and overall mean COVID-19 PCP at the Registration and Clinic Preparation Areas (F value =5.97; *p* < 0.0001). Bartlett's test for equal variances shows that there is a significant relationship between health facility and overall mean COVID-19 PCP at the Registration and Clinic Preparation Areas (chi^2^ (5) = 76.3324; *p* value <0.0001). The highest mean COVID-19 PCP at the Registration and Clinic Preparation Areas was recorded in H008 representing 2.63 (95%CI: 2.57–2.70) with a standard deviation of 0.31. The lowest mean COVID-19 PCP at the Registration and Clinic Preparation Areas was recorded in H001 representing 2.35 (95%CI: 2.31–2.40) with a standard deviation of 0.19.  Table 3 shows the comparative analysis of health facility and overall mean COVID-19 PCP at the Registration and Clinic Preparation Areas.

### 3.3. PCP at the Filing Area

In this section, the focus was on the filing areas. Respondents were asked about various practices related to COVID-19 protocols in this specific area. Regarding access restrictions, approximately 861 (69.1%) of participants affirmed that filing areas were restricted and accessible only to authorized Health Records and Information Officers (HRIO). On the other hand, 303 (23.9%) mentioned that access was sometimes restricted, while 104 (8.2%) stated that it was never restricted. In terms of HRIOs wearing hand gloves, 538 (42.4%) claimed that HRIOs in the filing area always had hand gloves, 402 (31.7%) said they sometimes had them, and 328 (25.9%) stated that HRIOs in the filing area did not have hand gloves at all.

Similar to hand gloves, respondents' answers about HRIOs wearing lab coats and hospital scrubs showed that 537 (42.4%) always had them, 411 (32.4%) sometimes had them, and 320 (25.2%) never had them. Regarding the use of 3-ply surgical face masks by HRIOs, 678 (53.5%) confirmed using them, 384 (30.3%) used them sometimes, and 206 (16.2%) never used them.

When asked if HRIOs sanitized and washed their hands with soap and water when entering and leaving filing areas, 879 (69.3%) responded affirmatively, 323 (25.5%) did it sometimes, and 66 (5.2%) never did. Concerning the decontamination of surfaces in contact with patient-level documents, 795 (62.7%) of respondents admitted doing it always, 100 (8%) never did it, and 373 (29.4%) seldom did it. Lastly, respondents were asked about the use of reminders, such as “wash hands before entry” and “no entry to unauthorized persons.” About 887 (70.0%) used reminders, 315 (24.8%) used them sometimes, and 66 (5.2%) never used these reminders. In summary, 739 (58.3%) of respondents always adhered to COVID-19 protocols in the filing area, 358 (28.3%) seldom adhered to them, and 170 (13.4%) did not adhere to them at all.

Similarly, the comparative analysis shows that there is significant variation in health facility and overall mean COVID-19 PCP at the filing area (F value =9.96; *p* < 0.0001). Bartlett's test for equal variances shows that there is a significant relationship between health facility and overall mean COVID-19 PCP at the filing area (chi^2^ (5) = 23.4388; *p* value =0.001). The overall mean COVID-19 PCP at the filing area ranged from 2.26 to 2.63 among the age groups.  Table 4 shows the comparative analysis of the health facility and overall mean COVID-19 PCP at the filing area.

### 3.4. PCP in the Handling of Health Records

The following set of questions focuses on the handling of health records. The first variable of interest was whether clinicians handled medical records while doffing personal protective equipment (PPE). Approximately 571 (45.0%) always handled records with PPE, 471 (37.1%) sometimes did, and 226 (17.8%) never handled medical records with PPE. About 740 (58.4%) of clinicians admitted that they created a safe place for keeping patients' files away from the room where clients are being seen. While 426 (33.6%) of clinicians sometimes created that safe space, 102 (8.0%) never did. In terms of Health Records and Information Officers (HRIOs) using hand gloves, 590 (46.5%) used them all the time, 449 (35.4%) seldom used them, and 229 (18.1%) never used them.

Regarding leaving patient charts/records in the patient's rooms, 378 (29.8%) always left them in the room, and 354 (27.9%) rarely left them in the room, while 536 (42.3%) never left charts/records in patient's rooms. For the demarcation between clean and dirty areas, approximately 486 (38.3%) either dictated clinical notes always or seldom dictated on the other side of the fence, while 296 (23.3%) never did that. The next question was whether ward rounds are conducted by at least two HCWs, with one providing patient care and the other clinical notes without touching anything on the ward so that health records will be considered uncontaminated and taken out without disinfection. About 543 (42.8%) admitted they did it always, 510 (40.2%) seldom did it, and 215 (17.0%) never did it. Regarding the handling of documents after the outbreak, whether they are placed in a container, left inside until the virus is considered unviable, or fumigated with formaldehyde, a little above 473 (37.3%) admitted they did it always, while 439 (34.3%) did it sometimes, and 356 (28.1%) never did it. In summary, 540 (42.5%) of respondents handled health records with caution always, 448 (35.3%) did this sometimes, and 280 (22.2%) did not adhere to these precautions.

Table 5 shows the comparative analysis of health facility and overall mean COVID-19 PCP in handling of health records. The analysis revealed that there is a significant variation in health facility and overall mean COVID-19 PCP in handling of health records (*F* value =12.73; *p* < 0.0001). Bartlett's test for equal variances shows that there is a significant relationship between health facility and overall mean COVID-19 PCP in handling of health records (chi^2^ (5) = 36.9567; *p* value <0.0001). The overall mean COVID-19 in handling of health records ranged from 1.99 to 2.40 among the age groups.

### 3.5. PCP at the Handling of Computers and Other Equipment

In this section, we focus on the handling of computers and other equipment. Among the HCWs who used electronic systems, 133 (10.5%) never ensured that shared computer equipment was cleaned and disinfected, while 425 (33.5%) seldom ensured it. However, a majority of participants, representing 710 (56%), always ensured that shared computer equipment was cleaned and disinfected.

The next question was about HCWs entering data and scanning records into a laptop computer left inside the dirty area and transmitting data via the Internet, a cable, or a USB stick and disinfecting it with chlorine before transferring it to another computer outside the dirty area. Interestingly, 469 (37%) of participants reported that they always followed this practice, 423 (33.4%) did it sometimes, and 376 (29.7%) never considered doing it.

The last variable in this section was whether HCWs entered data with a personal digital assistant kept inside a plastic cover and disinfected it with chlorine before taking it out or transmitted data via Bluetooth or e-mail. About 382 (30.2%) of respondents admitted they always followed this procedure, a little above 331 (26%) did it sometimes, and the majority of HCWs, representing 554 (43.7%), never attempted to do it.

To summarize, 520 (41.0%) of respondents always handled computers and other equipment with specific adherence to COVID-19 PCP. Approximately 393 (31.0%) respondents did this sometimes, while 355 (28.0%) did not handle computers and other equipment with specific adherence to COVID-19 precautions.

Table 6 shows the comparative analysis of health facility and overall mean COVID-19 PCP in handling of computers/equipment. The analysis revealed that there is a significant variation in health facility and overall mean COVID-19 PCP in handling of computers/equipment (F value =20.00; *p* < 0.0001). Bartlett's test for equal variances shows that there is a significant relationship between health facility and overall mean COVID-19 PCP in handling of computers/equipment (chi^2^ (5) = 30.1798; *p* value <0.0001). The overall mean COVID-19 PCP in handling of computers/equipment ranged from 1.99 to 2.40 among the age groups.

## 4. Discussion

### 4.1. Background Characteristics of Respondents

The background characteristics of the respondents in this study indicate that the majority were females, belonging to the young adult age group. Additionally, a significant number of participants had worked in their respective roles for 0–5 years, primarily in nursing positions. The study included a diverse range of healthcare professionals, such as pharmacists, medical assistants, nurses, midwives, public health nurses, community health nurses, nutrition officers, disease control officers, biostatisticians/district/hospital health information officers, dispensary technicians, and laboratory technologists/technicians, among others. The high representation of nurses among the respondents is notable, which reflects their vital role in the healthcare system. Moreover, a considerable proportion of the participants held bachelor's degrees, which is a common educational qualification required for securing good jobs in the healthcare sector in Ghana. The study also revealed that over half of the respondents had received training on COVID-19, indicating their exposure to information and guidelines related to the pandemic. This finding is consistent with other studies that have highlighted the importance of providing training to healthcare professionals in managing COVID-19 cases effectively and safely [26–29]. Research conducted by [26, 27] aligns with the current study's demographic profile findings, indicating that young adult female healthcare professionals, particularly in nursing, medicine, and dentistry, are prominently represented. Furthermore, this study and others suggest that healthcare professionals who receive reliable information and continuous training on personal protective equipment (PPE) usage are more likely to adhere to COVID-19 safety protocols, particularly when handling patients' health records and information management.

The background characteristics of the respondents in this study indicate a predominance of young adult females, especially in nursing roles, with a significant number having received COVID-19 training. Ensuring that healthcare professionals are well-informed and adequately trained on COVID-19 safety measures is crucial in promoting compliance with protocols and enhancing patient care and safety.

### 4.2. PCP at the Registration and Clinic Preparation Areas

The findings related to infection control practices (PCP) at the registration and clinic preparation areas are encouraging, as they indicate that most healthcare provider participants (HCP-PR) in this study demonstrated adherence to COVID-19 precautions. The majority of respondents reported wearing face masks, sanitizing their hands regularly, and disinfecting various high-touch surfaces, such as computer keyboards, mice, door handles, preparation tables, and pens before and after use. These practices reflect HCP-PR's awareness of their crucial role in combating the COVID-19 pandemic and their understanding of the higher risk they face of contracting the virus while on the job, as supported by other studies [19, 30, 31].

The World Health Organization (WHO) has established several protocols for preventing the spread of COVID-19, and one of the key measures is the consistent use of face masks by healthcare workers. This practice has been shown to protect healthcare workers, including HCP-PR, during interactions with patients during registration, medical record preparation, and admission. Additionally, the provision of required personal protective equipment, such as face shields and masks, to staff posted in the triage examination area is essential for ensuring their safety [32]. Previous studies referenced [33, 34] also demonstrate the importance of hand hygiene in infection control. These studies reported that the majority of healthcare providers routinely used alcohol hand rubs and regularly cleaned their hands with soap and water. Moreover, a significant proportion of healthcare providers used face shields or masks regularly, further contributing to infection control measures. Frequent hand washing and hand rubbing are commonly reported practices in hospitals, as emphasized by other studies [20, 35–37], which align with the findings of this study.

Overall, the consistent findings across different studies and the current study support the notion that HCP-PR are actively implementing crucial infection control measures at the registration and clinic preparation areas. These practices are vital in preventing the spread of COVID-19 and protecting both healthcare workers and patients. However, continuous education, training, and reinforcement of these infection control practices are essential to ensure sustained compliance and to further enhance the safety and well-being of healthcare providers and patients alike.

### 4.3. PCP at the Filing Area

The findings from the study regarding infection control practices (PCP) at the filing area among healthcare provider participants (HCP-PR) are a little over four out of every seven HCP-PR always comply with COVID-19 prevention and control practices. This level of compliance may be attributed to the nature of the tasks performed by HCP-PR in the filing area. HCP-PR in this area handles returned patient folders, sort and file them, dispense medication prescribed by doctors, and review laboratory reports. These patient folders travel from various healthcare providers working in the facility, which creates the possibility of virus transmission. As indicated by other studies [38, 39], the awareness of the potential virus transmission through these files could be a motivating factor for HCP-PR to adhere to infection control protocols.

The use of 3-ply surgical face masks, regular hand sanitization or hand washing with soap and water, and the implementation of reminders, such as handwashing before entry and restricting unauthorized access, are measures reported in the referenced studies to promote infection control practices in similar settings. However, challenges related to the correct donning and doffing of personal protective equipment (PPE) have been noted in some studies [38, 39]. These challenges may stem from the availability of PPE, limitations in knowledge, and behavioral issues among healthcare providers. Instances of PPE reuse and inadequate compliance with PPE usage have been reported in some settings [21, 38, 39], while others have indicated unsatisfactory PPE usage rates, as highlighted by Aveng-Nkansah et al. [40]. Comparing the findings of this study to the referenced study [40], the level of compliance with infection control protocols at the filing area appears to be slightly higher in the current study. However, both studies demonstrate room for improvement in infection control practices, particularly in areas related to correct PPE usage.

Overall, the findings suggest that while many HCP-PR in the filing area are following infection control protocols, there are still challenges to address, such as proper PPE usage and compliance. Efforts should be made to enhance education and training on correct PPE usage and reinforce the importance of consistent adherence to infection control practices to further reduce the risk of COVID-19 transmission in healthcare settings. Additionally, ensuring a steady supply of PPE and addressing knowledge gaps among healthcare providers can contribute to improved infection control practices in the filing area and beyond.

### 4.4. PCP in the Handling of Health Records

The handling of health records is an essential aspect of healthcare services, and healthcare providers regularly interact with patient information throughout the care process. Patient records are collected during registration, and investigation reports from the laboratory are shared with authorized providers to facilitate the provision of healthcare services to patients. However, the findings of this study indicate that the majority of healthcare provider participants (HCP-PR) did not always adhere to infection control practices (PCP) when handling patients' records. This nonadherence to PCP in handling health records is a matter of concern, as it poses a potential risk for the spread of COVID-19 and other infections within healthcare settings. The research conducted by Savio et al. [41] is consistent with the findings of this study, highlighting the challenges healthcare professionals face in complying with the use of personal protective equipment (PPE) when working with COVID-19 patients. PPE is a crucial element in preventing the transmission of infections, including COVID-19, and its correct usage is vital for the safety of both healthcare providers and patients.

The nonadherence to PCP in handling health records may be attributed to various factors, including the discomfort associated with wearing PPE for extended periods, potential shortages of PPE, or a lack of awareness about the importance of adhering to infection control measures. Additionally, the hectic nature of healthcare settings and the pressure to provide prompt care to patients might also contribute to lapses in adhering to PCP. Addressing these challenges requires comprehensive strategies, including ongoing education and training on infection control practices, ensuring a consistent supply of PPE, and creating a culture of safety and accountability within healthcare facilities. Healthcare providers need to be continuously reminded of the importance of following PCP, especially when handling patients' health records, to minimize the risk of infection transmission.

Overall, promoting a robust infection control culture and providing the necessary resources and support to healthcare providers can help improve adherence to PCP and enhance the safety of both healthcare workers and patients. It is crucial for healthcare organizations to prioritize the implementation of effective infection control practices and ensure that healthcare providers are adequately equipped to protect themselves and their patients during the handling of health records and other patient-related activities.

### 4.5. PCP at Handling Computers/Equipment

The handling of computers and equipment is an important aspect of infection control practices (PCP) in healthcare settings, especially during the COVID-19 pandemic. The survival of viruses on surfaces is influenced by various factors, such as the type of surface, relative humidity, temperature, and virus strain [15]. To prevent the spread of the virus throughout healthcare facilities, healthcare providers (HCPs) are strongly encouraged to regularly disinfect surfaces that come into contact with humans, including laptops, mice, keyboards, and other equipment used with patients [15]. Multiple studies have highlighted the significance of disinfecting equipment and surfaces as an essential infection control practice to prevent the transmission of SARS-CoV-2, as the virus can remain stable on surfaces for several days [24, 42]. Proper disinfection of shared computer equipment is crucial to minimize the risk of cross-contamination between patients and healthcare workers. To reduce the risk of transmission further, some studies suggest that healthcare providers should minimize the physical handling of equipment, such as computers, in clinical environments [25]. This can be achieved by implementing strategies that limit direct contact with equipment, thus reducing the potential for virus transmission.

The findings of the current study indicate that a disappointingly low proportion of HCP-PR consistently adhere to COVID-19 PCP when handling computers and other equipment. This calls for improvement in infection control practices to minimize the spread of infections within healthcare facilities. However, it is encouraging that more than half of HCP-PR consistently ensured that shared computer equipment was cleaned and disinfected. This finding aligns with a previous study [18, 19], affirming that hospital computers are regularly disinfected with appropriate agents before and after use. While the current study did not provide a specific proportion of HCPs who comply with the disinfection aspect of infection control practices, it underscores the importance of ensuring proper disinfection measures are in place. The consistent cleaning and disinfection of shared computer equipment can significantly contribute to reducing the risk of infection transmission in healthcare settings.

The handling of computers and equipment is a critical aspect of infection control in healthcare settings, particularly during the COVID-19 pandemic. Proper disinfection practices, along with strategies to minimize physical handling, are essential in preventing the spread of infections. Healthcare facilities should prioritize education and training for healthcare providers to ensure the consistent and effective implementation of infection control practices in handling computers and equipment. By doing so, healthcare facilities can create a safer environment for both patients and healthcare workers.

The analysis demonstrates noteworthy variations in healthcare providers' perceived adherence to COVID-19 patient care practices (PCP) within health records and information management across various thematic areas. Through statistical tests and mean values, the study offers insights into the diverse and differing levels of performance in these practices. The findings highlight specific health facilities that stand out due to their distinctive adherence to COVID-19 PCP, shedding light on the variability and performance levels specific to each facility.

This study provided a comprehensive indication of the importance of adherence to COVID-19 PCP, particularly in the context of healthcare providers managing health records and information in Ghana. There appears to be limited literature on the long-term sustainability of COVID-19 PCP. Furthermore, studies investigate the sustainability of COVID-19 PCP in health records management beyond the immediate pandemic response, considering factors such as cost-effectiveness, scalability, and integration into routine healthcare workflows is crucial.

## 5. Conclusions

The background characteristics of the respondents in this study indicate a significant representation of young adult females, particularly in nursing roles, with a substantial number having received COVID-19 training. Ensuring that healthcare professionals are well-informed and adequately trained on COVID-19 safety measures is crucial in promoting compliance with protocols and enhancing patient care and safety.

The findings related to infection control practices at the registration and clinic preparation areas are encouraging, with a majority of healthcare provider participants (HCP-PR) demonstrating adherence to COVID-19 precautions. This indicates that HCP-PR are aware of their role in combating the pandemic and the higher risk they face while on the job. Proper use of personal protective equipment (PPE), frequent hand sanitization, and surface disinfection are vital practices to prevent the spread of infections in healthcare settings.

In the filing area, a little over four out of every seven HCP-PR always comply with COVID-19 prevention and control practices. While this level of compliance is positive, there are still challenges related to correct PPE usage and adherence to infection control protocols that need to be addressed. Education, training, and ensuring a steady supply of PPE are crucial in improving infection control practices in this area.

The handling of health records is an area of concern, as the majority of HCP-PR did not always adhere to infection control practices. Nonadherence to PCP in this context poses a potential risk for the spread of COVID-19 and other infections within healthcare settings. Proper use of PPE and continuous reinforcement of infection control protocols are necessary to minimize the risk of transmission during the handling of health records.

Similarly, handling computers and equipment also presents challenges in infection control. Disinfection of shared computer equipment is essential to minimize the risk of cross-contamination. Although there is room for improvement in adherence to infection control practices, encouragingly, more than half of HCP-PR consistently ensured that shared computer equipment was cleaned and disinfected.

Analysis reveals diverse adherence to COVID-19 PCP in health records. Statistical tests show variable performance, highlighting standout health facilities [43].

## 6. Policy Implications

1. Healthcare facilities should prioritize training and education for healthcare professionals, especially in infection control practices related to COVID-19. Continuous reinforcement and updates on protocols are essential to ensure adherence.
2. Availability and access to adequate PPE are critical. Healthcare facilities should ensure a steady supply of PPE and address any shortages to facilitate compliance with infection control practices.
3. Implementing reminders and protocols, such as frequent hand sanitization and surface disinfection, can help foster a culture of safety and encourage consistent adherence to infection control practices.
4. Healthcare organizations should develop comprehensive infection control policies and guidelines specific to different areas within healthcare facilities, such as registration, clinic preparation, filing, and handling of health records and equipment.
5. Regular monitoring and assessment of infection control practices are essential to identify areas for improvement and to address any challenges in compliance.
6. Collaboration and communication among healthcare providers, administrators, and policymakers are crucial to promote a culture of safety and to ensure a coordinated and effective approach to infection control.

By implementing these policy implications, healthcare facilities can enhance infection control practices, reduce the risk of COVID-19 transmission, and create a safer environment for both healthcare workers and patients.

## Figures and Tables

**Table 1:** Descriptive statistics, scale, and item reliability test.

Dimensions	Number of items	Health professionals (*n* = 1268)			Cronbach's alpha coefficient	Content validity index	Content validity ratio
		Mean	SD	Mean values under 95% CI			
Overall tool	50	1.46	0.26	[1.45–1.48]	0.73	88.0	100.0
BQ	18	1.32	0.28	[1.30–1.33]	0.86		
CAQ	9	1.49	0.37	[1.47–1.52]	0.78		
CBQ	7	1.55	0.46	[1.53–1.58]	0.78		
CCQ	7	1.79	0.48	[1.77–1.82]	0.74		
CDQ	3	1.87	0.63	[1.83–1.90]	0.73		
CEQ	6	1.17	0.30	[1.15–1.19]	0.82		

CI, confidence interval; SD, standard deviation; BQ, general adherence to COVID-19 protocol (Health Records and Information Management Personnel); CAQ, registration area requirements/clinic preparation areas; CBQ, filing area; CBQ, handling of health records; CDQ, handing of computers/equipment; CEQ, patient education always.

**Table 2:** Sociodemographic characteristics of respondents.

Variables	Number of respondents (*n* = 1268)	Percentage
Sex		
Male	552	43.6
Female	715	56.4
Age		
20–29	560	44.2
30–39	545	43.0
40–49	126	9.9
50+	19	1.5
No response	18	1.4

Source: Survey, 2022.

**Table 3:** Comparative analysis of health facility and overall mean COVID-19 PCP at the Registration and Clinic Preparation Areas.

Facility code	Mean	Standard deviation	95% confidence interval		*F* value; *p* value
			Lower limit	Upper limit	
H001	2.35	0.19	2.31	2.40	5.97; *p* < 0.0001^*∗∗*^
H002	2.47	0.23	2.41	2.52	
H003	2.47	0.27	2.41	2.53	
H004	2.59	0.32	2.53	2.64	
H005	2.54	0.30	2.48	2.60	
H006	2.51	0.32	2.45	2.56	
H007	2.52	0.29	2.46	2.58	
H008	2.63	0.31	2.57	2.70	

Bartlett's test for equal variances: chi^2^ (5) = 76.3324; *p* value <0.0001^*∗∗*^. Statistically significant at *p* value <0.05^*∗*^ and *p* value <0.0001^*∗∗*^

**Table 4:** Comparative analysis of health facility and overall mean COVID-19 PCP at the filing area.

Facility code	Mean	Standard deviation	95% confidence interval		*F* value; *p* value
			Lower limit	Upper limit	
H001	2.34	0.30	2.28	2.40	9.96; *p* < 0.0001^*∗∗*^
H002	2.46	0.36	2.39	2.53	
H003	2.26	0.41	2.17	2.35	
H004	2.56	0.35	2.49	2.62	
H005	2.51	0.39	2.44	2.57	
H006	2.52	0.34	2.45	2.58	
H007	2.35	0.37	2.28	2.43	
H008	2.63	0.41	2.57	2.68	

Bartlett's test for equal variances: chi^2^ (5) = 23.4388; *p* value =0.001^*∗*^. Statistically significant at *p* value <0.05^*∗*^ and *p* value <0.0001^*∗∗*^

**Table 5:** Comparative analysis of health facility and overall mean COVID-19 PCP in handling of health records.

Facility code	Mean	Standard deviation	95% confidence interval		*F* value; *p* value
			Lower limit	Upper limit	
H001	1.99	0.42	1.94	2.05	12.73; *p* < 0.0001^*∗∗*^
H002	2.11	0.45	2.03	2.19	
H003	2.34	0.56	2.28	2.40	
H004	2.37	0.40	2.30	2.44	
H005	2.05	0.40	1.98	2.12	
H006	2.30	0.43	2.23	2.36	
H007	2.12	0.47	2.03	2.20	
H008	2.40	0.37	2.33	2.48	

Bartlett's test for equal variances: chi^2^ (5) = 36.9567; *p* value =0.0001^*∗∗*^. Statistically significant at *p* value <0.05^*∗*^ and *p* value <0.0001^*∗∗*^.

**Table 6:** Comparative analysis of health facility and overall mean COVID-19 PCP in handling of computers/equipment.

Facility code	Mean	Standard deviation	95% confidence interval		*F* value; *p* value
			Lower limit	Upper limit	
H001	1.99	0.39	1.77	1.92	20.00; *p* < 0.0001^*∗∗*^
H002	2.11	0.51	1.65	1.82	
H003	2.34	0.39	2.09	2.29	
H004	2.37	0.45	2.36	2.52	
H005	2.05	0.44	1.78	2.01	
H006	2.30	0.43	2.27	2.42	
H007	2.12	0.53	2.15	2.33	
H008	2.40	0.48	2.31	2.50	

Bartlett's test for equal variances: chi^2^ (5) = 30.1798; *p* value =0.0001^*∗∗*^. Statistically significant at *p* value <0.05^*∗*^ and *p* value <0.0001^*∗∗*^.

## Data Availability

The data that support the findings of this study are included in this article. Anyone who is interested in the raw data may contact the corresponding author.
